# Serum Cystatin C for the Diagnosis of Acute Kidney Injury in Patients Admitted in the Emergency Department

**DOI:** 10.1155/2015/416059

**Published:** 2015-06-15

**Authors:** Cristina Bongiovanni, Laura Magrini, Gerardo Salerno, Chiara Serena Gori, Patrizia Cardelli, Mina Hur, Marco Buggi, Salvatore Di Somma

**Affiliations:** ^1^Department of Emergency Medicine, Medical-Surgery Sciences and Translational Medicine, School of Medicine and Psychology, Sant'Andrea Hospital, Sapienza University of Rome, 00185 Rome, Italy; ^2^Department of Molecular and Clinical Medicine, School of Medicine and Psychology, Sant'Andrea Hospital, Sapienza University of Rome, 00185 Rome, Italy; ^3^Department of Laboratory Medicine, Konkuk University School of Medicine, Seoul 143-701, Republic of Korea; ^4^Nursing Science, School of Medicine and Psychology, Sant'Andrea Hospital, Sapienza University of Rome, 00185 Rome, Italy

## Abstract

*Background*. Early diagnosis of acute kidney injury (AKI) at emergency department (ED) is a challenging issue. Current diagnostic criteria for AKI poorly recognize early renal dysfunction and may cause delayed diagnosis. We evaluated the use of serum cystatin C (CysC) for the early and accurate diagnosis of AKI in patients hospitalized from the ED. *Methods*. In a total of 198 patients (105 males and 93 females), serum CysC, serum creatinine (sCr), and estimated glomerular filtration rate (eGFR) were calculated at 0, 6, 12, 24, 48, and 72 hours after presentation to the ED. We compared two groups according to the presence or absence of AKI. *Results*. Serial assessment of CysC, sCr, and eGFR was not a strong, reliable tool to distinguish AKI from non-AKI. CysC > 1.44 mg/L at admission, both alone (Odds Ratio = 5.04; 95%CI 2.20–11.52; *P* < 0.0002) and in combination with sCr and eGFR (Odds Ratio = 5.71; 95%CI 1.86–17.55; *P* < 0.002), was a strong predictor for the risk of AKI. *Conclusions*. Serial assessment of CysC is not superior to sCr and eGFR in distinguishing AKI from non-AKI. Admission CysC, both alone and in combination with sCr and eGFR, could be considered a powerful tool for the prediction of AKI in ED patients.

## 1. Introduction

Acute kidney injury (AKI) is a common complication in hospitalized patients [1–4]. Despite progress in medical care, it is still associated with increased morbidity, mortality, length of hospital stay, costs, and postacute care resource utilization [4, 5]. AKI is defined as a rapid (hours to days) decrease in renal excretory function with an accumulation of products of nitrogen metabolism, such as creatinine (sCr), urea, and other clinically unmeasured waste products [6]. In routine clinical practice, sCr is used to estimate renal function and, accordingly, as a marker for diagnosing and staging of AKI [6, 7]. The risk, injury, failure, loss of function, end-stage renal disease (RIFLE) [8, 9], and the Acute Kidney Injury Network (AKIN) [10] criteria provide a uniform definition of AKI and have become the standard for diagnostic criteria [11].

It may be, however, problematic to diagnose AKI in the patients presented to the emergency department (ED). Serial assessment of sCr and monitoring of urine output for several days are necessary for the diagnosis of AKI. In the ED, however, the baseline sCr level is often unknown and placement of a urinary catheter may not be indicated, leading to a delay in diagnosis and, therefore, in adequate therapy [12]. Moreover, despite evidence of nephron damage, no diagnostic changes in sCr level may be detected in the cases of subclinical AKI [13]. As a consequence, there is a need for new biomarkers that could aid in early diagnosis and prediction of AKI and, ideally, could be a tool for discriminating prerenal, intrarenal, or postrenal AKI from one another, helping in identifying the aetiologies of AKI and predicting and monitoring the response to AKI interventions [14, 15].

Serum cystatin C (CysC) is freely filtered through the glomerular membrane and completely reabsorbed and metabolized by the proximal tubular cells without secretion [16, 17]. CysC concentration is affected neither by inflammation, fever, and/or outside agents nor by muscle mass, gender, or age [18–21]. Previous studies reported contradictory results on the accuracy of CysC in the early diagnosis of AKI and precluded its widespread use as a calculation of estimated glomerular filtration rate (eGFR) in clinical practice [17, 22–29]. In this study, we wanted to investigate the potential use of CysC for the early and accurate diagnosis of AKI, especially in the patients hospitalized from the ED. We questioned which would be preferable between single and serial measurements of CysC, in comparison with sCr and eGFR.

## 2. Patients and Methods

### 2.1. Study Population

During the period from November 2008 to April 2009, a total of 203 patients were consecutively enrolled from the ED of Sant'Andrea Hospital in Rome. Except for five patients with incomplete data, 198 patients (105 males and 93 females) were finally included in this study. Excluded were the patients with a history of marked chronic renal insufficiency (usual sCr ≥ 3.0 mg/dL) or urothelial malignancy; on dialysis or renal replacement therapy (RRT) (either acute or chronic); in imminent need of dialysis or RRT at enrolment; or with a participation history in any interventional clinical study within the previous 30 days. This prospective clinical trial was designed following the criteria of the Declaration of Helsinki and approved by the ethical committee of the hospital. Written informed consent was obtained by each patient prior to enrolment.

**Figure 1:**
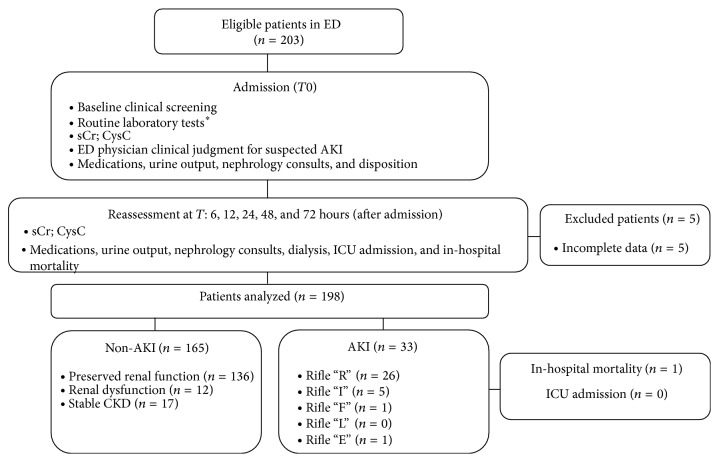
Flow diagram of the study design.

In all the patients, past medical history and demographic data were recorded at admission, and routine physical and laboratory work-ups were performed. Each patient was treated on the basis of the formulated diagnosis at discretion of the treating physician, and therapy was carefully recorded. There were no documented prestudy sCr levels available for any of the enrolled patients. We calculated a baseline value for sCr by excluding patients with preexisting chronic kidney disease (CKD) and by calculating the baseline sCr using the median values (interquartile range, IQR) measured during hospital stay, the lowest three of them, that is 0.9 (IQR 0,70–1.20) mg/dL. Renal dysfunction, the development of oliguria, the need for a nephrology consultation, initiation of dialysis or RRT (following international guidelines) [11], intensive care unit (ICU) admission, and mortality were recorded during the hospitalization (Figure 1).

### 2.2. Clinical Adjudication

According to the RIFLE criteria, all patients were divided into two groups: AKI (*n* = 33, 16.7%) and non-AKI (*n* = 165, 83.3%) groups [9, 10]. The renal function classification and patients' assignment into the two groups were performed and confirmed, according to RIFLE criteria, by the consensus of the Nephrologists of the study group who were blinded to the biomarker results. AKI was defined with a new onset of at least 1.5-fold increase of sCr values from baseline or a eGFR decrease >25%. The non-AKI group included patients with normal kidney function (NF), nonprogressive CKD, and prerenal azotemia (PreR). NF was defined as a baseline eGFR > 60 mL/min per 1.73 m^2^ and no increase in sCr during the hospitalization. Nonprogressive CKD was defined as a sustained and unchanging decrease in eGFR that met criteria for CKD (eGFR < 60 mL/min per 1.73 m^2^) and persisted for more than 3 months before admission [11]. PreR was defined as a new-onset increase in sCr that resolved within 48 hours and returned to the baseline NF level.

### 2.3. Measurement of CysC, sCr, and eGFR

Renal function was assessed by measuring sCr and CysC. Serial blood samples were obtained at 0, 6, 12, 24, 48, and 72 hours from presentation to the ED. After centrifugation at 3,600 rpm for 15 minutes, serum samples were stored at −20°C until used. CysC was measured by particle enhanced immunonephelometric assay (N Latex Cystatin C, Siemens, Marburg, Germany), and its measuring range was 0.76 mg/L to 1.44 mg/L. The results of CysC were blinded to the medical staffs during the study and did not affect the management of patients. sCr was measured by enzymatic assay (Vitros Crea; Ortho Clinical Diagnostics, High Wycombe, UK), and its normal range was 0.8 to 1.5 mg/dL. The biomarker assays were performed following manufacturer's instructions. As recommended by Kidney Disease Outcome Quality Initiative (KDOQI) guidelines, eGFR was calculated using the modification of diet in renal disease (MDRD) formula [30–34].

### 2.4. Statistical Analysis

The results were expressed as mean ± standard deviation (SD) for normally distributed values or median and interquartile range for the variables without normal distribution. The normality of data distribution was checked with D'Agostino and Pearson normality test. Gender, age, height, weight, BMI, and comorbidities were compared between AKI and non-AKI groups, using the chi-square test for categorical variables and one-way analysis of variance for continuous variables. CysC, Cr, and eGFR were compared between AKI and non-AKI groups using Mann-Whitney *U* test and Kruskall-Wallis test. A logistic univariate model was used to select the variables most predictive of AKI development. Odds ratios (OR) with 95% confidence interval (CI) were calculated using the Wald method. A receiver operating characteristic (ROC) curve was utilized to determine the ability of CysC, sCr, and eGFR at *T*0 to predict AKI. The area under curve (AUC) indicated the predictive value of each biomarker (*P* value < 0.05 was considered statistically significant).

## 3. Results

Patients' characteristics are presented in Table 1. There was no statistical difference in the distribution of gender, age, and body mass index (BMI) between the two groups. Except for chronic obstructive pulmonary disease (*P* < 0.001), the distribution of comorbidities did not differ between the two groups. Mean infusion volume was 950 milliliters/daily, and mean urine output was 1250 milliliters/daily with a mean fluid balance of −300 milliliters in the first 24 hours.

**Figure 2:**
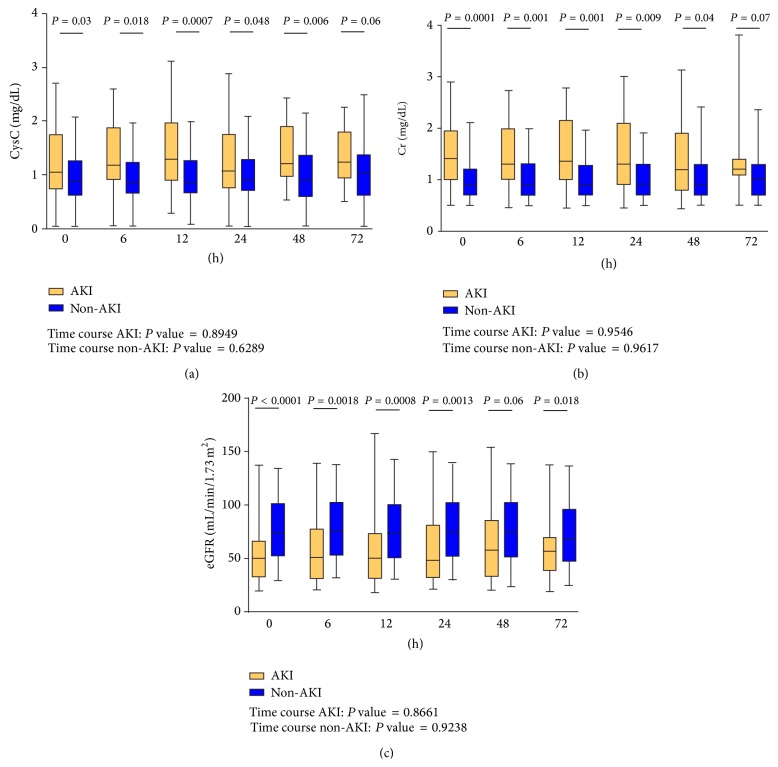
Serial comparison of CysC, sCr, and eGFR between AKI and non-AKI groups.

Figure 2 shows the serial comparison of CysC, sCr, and eGFR between AKI and non-AKI groups. When considering serial measurements and the comparison of AKI versus non-AKI, CysC and sCr showed significant differences between the two groups at each measurement time until 48 hours. eGFR also showed such a significant difference from admission until 24 hours.

When only AKI group was evaluated, all the three markers, CysC, sCr, and eGFR, did not show any statistical difference, according to the time course, and the same was for non-AKI group. In patients with AKI the 1.5-fold increase of sCr was at a mean of 37.6 ± 23 (hours ± SD) after admission in ED.

In univariate analysis, CysC, sCr, and eGFR at admission were compared for the prediction of AKI development, and, moreover, their combination was evaluated. Admission CysC level > 1.44 mg/L was strongly related to the increased risk of AKI development (OR = 5.04; 95% CI, 2.20–11.52; *P* < 0.0002). sCr level > 1.5 mg/dL (OR = 2.84; 95% CI, 1.26–6.37; *P* < 0.01) and eGFR < 60 mL/min/1.73 m^2^ (OR = 3.33; 95% CI, 1.50–7.38; *P* < 0.003) were also related to the increased risk of AKI, but not strongly. The best predictive model for AKI development was the combined use of CysC, sCr, and eGFR at admission (OR = 5.71; 95% CI, 1.86–17.55; *P* < 0.002); the combination of CysC + sCr had an OR = 3.48 (95% CI, 1.70–7.01, *P* < 0.01), and the combination of CysC + eGFR had an OR = 4.35 (95% CI, 1.68–8.12, *P* < 0.02) (Table 2).

A ROC curve analysis was performed for CysC, sCr, and eGFR at admission. We evaluated ROC prognostic value of the markers both alone and in combination. The AUC of CysC compared to sCr and to eGFR showed a higher predictive power (CysC = 0.72, *P* < 0.02; sCr = 0.70, *P* < 0.01; eGFR = 0.71, *P* < 0.01). The AUC of CysC + sCr and of CysC + eGFR showed a good high diagnostic value for AKI (resp., AUC 0.70, *P* < 0.0004, and AUC 0.70, *P* < 0.0003) but lower than that of CysC alone.

## 4. Discussion

The early diagnosis of AKI in ED is a challenging issue. The current diagnosis of AKI is based on the serial measurement of sCr and/or the monitoring of urinary output [6, 7]. sCr is greatly influenced by several renal and nonrenal factors, including changes in muscle mass, tubular secretion, extracellular fluid volume expansion, malnutrition, race, age, gender, and medications [35–37]. Moreover, it is difficult to carefully collect and monitor urine output in ED. These factors could hamper the detection of sCr increase despite significant kidney injury and delay the diagnosis of AKI [38]. Many biomarkers have been studied to solve this problem and, among them, CysC has got an increasing attention recently.

CysC is produced at a constant rate by all nucleated cells and its concentration is not influenced by age, sex, height, and body composition. Accordingly, CysC concentration reflects only the balance of its primary physiological determinants: cellular generation, renal filtration, and subsequent renal degradation [16–21]. An increased CysC concentration identifies early deviations in eGFR and, subsequently, a “preclinical” state of kidney dysfunction that is not detected with sCr or eGFR [21].

The previous studies on CysC were mostly conducted in specific clinical settings (i.e., department of nephrology, coronary care unit, and ICU) with relatively homogeneous and selected study populations. Moreover, the literature data are based on only one measurement of CysC at baseline. In the ED, such studies on CysC have been limited. Nickolas et al. [13] demonstrated that urinary CysC had a slight predictive power on diagnosis of AKI in ED. In 2010 Soto et al. published a study conducted in a nonsurgical ED, evaluating serum and urinary Cr and CysC at several times from ED presentation in 800 consecutive patients. They concluded on behalf of CysC as a better and earlier predictive biomarker of AKI than sCr [20]. Differently, in our study, a smaller and more heterogeneous sample was evaluated, and they were both surgical and nonsurgical patients. As a matter of fact, our results showed that there are statistically significant differences among the two groups (AKI versus non-AKI) for CysC values at several times (admission, 6 hours, 12 hours, and 48 hours) but the same results were found for sCr values (admission, 6 hours, 12 hours, 24 hours, and 48 hours) and eGFR values (admission, 6 hours, 12 hours, and 24 hours) confirming that the traditional methods used, nowadays, are enough for the detection of AKI.

However, their serial assessment did not show any significant difference according to the time course in each group [20]. Compared with the traditional markers of sCr and eGFR, CysC did not show any advantage for the prediction of AKI (Figure 2). Noticeably, we found that CysC > 1.44 mg/L at admission had a greater power in predicting AKI (OR = 5.04) than sCr (OR = 2.84) and eGFR (OR = 3.33). In addition, the combined use of CysC, sCr, and eGFR at admission showed the best result for the prediction of AKI (Table 2).

ROC curve analysis confirmed the higher diagnostic power of CysC, not only when combined with the other two markers, but also alone; this indicates probably a more diagnostic accuracy of CysC in comparison with sCr and eGFR, also if the serial measurements showed the same statistical significance in the AKI versus non-AKI group for all the three markers studied.

This study is limited in that relatively smaller and more heterogeneous patients were enrolled and they were both surgical and nonsurgical patients that could have caused a wide variability of measured data, especially for Cr. Moreover, patients with Cr > 3 mg/dL were excluded from the study, and the patients were not followed up. The ED population is one of the most difficult groups in which to define the baseline time with respect to the development of the clinical problem. However, in the ED, the diagnosis and risk prediction of AKI are made in these heterogeneous populations without enough data fulfilling the diagnostic criteria, and our study population may simulate the general population afferent to the ED for various acute conditions.

In conclusion, we investigated the potential use of CysC for the diagnosis of AKI in the patients presented to the ED. Our data demonstrates that the serial assessment of CysC does not provide additional value for the diagnosis of AKI, as well as Cr and eGFR. On the contrary, an elevated CysC concentration is associated with a higher risk of developing AKI in the general population afferent to the ED. Measurement of CysC at admission, alone or combined with Cr and eGFR, is a preferable option for the prediction of AKI in the ED. Larger studies are awaited to support the present findings and to define the specific role of serum CysC in AKI.

## Figures and Tables

**Table 1:** Patients' characteristics.

	Total	AKI group	Non-AKI group
	(*n* = 198)	(*n* = 33, 16.7%)	(*n* = 165, 83.3%)
Male/female (*n*)	105/93	18/15	81/84
Age (years)	74 ± 13.6	73 ± 14.0	78 ± 10.4
Height (cm)	165.7 ± 8.8	166.0 ± 8.0	166.0 ± 9.0
Weight (kg)	72.9 ± 15.7	74.0 ± 17.0	73.0 ± 15.0
BMI (kg/m^2^)	26.5 ± 5.1	26.9 ± 5.4	26.4 ± 5.0

Comorbidities (*n*, %)			
Coronary artery disease	53 (26.8)	8 (24.2)	45 (27.3)
Hypertension	125 (63.1)	23 (69.7)	99 (60.0)
Dyslipidemia	26 (13.1)	4 (12.1)	21 (12.7)
Arrhythmia	55 (27.8)	10 (30.3)	45 (27.3)
Pacemaker	14 (7.1)	4 (12.1)	8 (4.8)
Valvulopathies	12 (6.1)	2 (6.1)	10 (6.1)
COPD	65 (32.8)	19 (57.6)	46 (27.9)^*∗*^
Diabetes	57 (28.8)	9 (27.3)	46 (27.9)
Chronic kidney disease	22 (11.1)	5 (15.2)	17 (10.3)
Anemia	18 (9.1)	4 (12.1)	13 (7.9)
Stroke/TIA	24 (12.1)	3 (9.1)	20 (12.1)
Pulmonary embolism	2 (1.0)	1 (3.0)	1 (0.6)

^*∗*^ *P* < 0.0001 versus AKI group.

Data are expressed as mean ± standard deviation or number (percentage).

AKI, acute kidney injury; BMI, body mass index; COPD, chronic obstructive pulmonary disease; TIA, transient ischemic attack.

**Table 2:** Univariate analysis of CysC, sCr, and eGFR at admission for the risk of acute kidney injury development.

	OR	95% CI	*P* value
CysC	5.04	2.20–11.52	0.0002
sCr	2.84	1.26–6.37	0.01
eGFR	3.33	1.50–7.38	0.003
CysC + sCr	3.48	1.70–7.01	0.01
CysC + eGFR	4.35	1.68–8.12	0.002
CysC + sCr + eGFR	5.71	1.86–17.55	0.002

CysC, cystatinC; eGFR, estimated glomerular filtration rate; sCr, serum creatinine; OR, odds ratio; CI, confidence interval.
